# Utilization of Tunnel Waste Slag for Cement-Stabilized Base Layers in Highway Engineering

**DOI:** 10.3390/ma17184525

**Published:** 2024-09-14

**Authors:** Junshuang Deng, Yongsheng Yao, Chao Huang

**Affiliations:** 1 Jiangxi Transportation Engineering Group Co., Ltd., Nanchang 330038, China; dengjs01@163.com; 2 College of Traffic & Transportation, Chongqing Jiaotong University, Chongqing 400074, China; 3 School of Civil Engineering, Central South University, Changsha 410075, China

**Keywords:** pavement, tunnel waste slag, recycled aggregates, cement-stabilized aggregate base, mechanical properties

## Abstract

The rapid expansion of highway infrastructure in the mountainous regions of China has led to a significant increase in tunnel construction, generating substantial amounts of tunnel waste slag. Concurrently, the development of transportation infrastructure has created a critical shortage of natural aggregates, necessitating the exploration of alternative sustainable sources. This study aimed to conduct a comprehensive evaluation of the physical and mechanical properties of tunnel waste slag and explore its potential for utilization in cement-stabilized base courses for highway engineering applications. The uniaxial compressive strength of the parent rock (tunnel waste slag) ranged from 81 MPa to 89 MPa in the desiccated state, indicating its suitability for use as a construction material. This study also determined the maximum dry density (2.432 g/cm^3^) and optimal moisture content (5.4%) of cement-stabilized mixtures incorporating recycled aggregates derived from tunnel waste slag. The splitting tensile strength of these mixtures at 28 days varied from 0.48 MPa to 0.73 MPa, demonstrating robust mechanical performance. Moreover, the unconfined compressive strength of these mixtures escalated from 7.0 MPa at 7 days to 11.0 MPa at 90 days, signifying a substantial enhancement in strength over time. These results validate the viability of utilizing tunnel waste slag in highway engineering and furnish valuable insights for designers, concrete manufacturers, and construction firms engaged in the development of cement-stabilized aggregate base courses.

## 1. Introduction

The rapid expansion of highway infrastructure in the mountainous regions of China has resulted in a substantial increase in the construction of new tunnels [1]. The excavation and tunneling processes associated with these highway projects produce considerable amounts of tunnel waste slag, presenting significant environmental challenges in terms of storage and disposal [2]. Concurrently, the development of highways and other transportation infrastructure necessitates vast quantities of natural aggregates, including sand and gravel, as fundamental constituents for concrete and pavement base course materials [3,4,5]. Data indicate that the consumption of aggregates for each kilometer of highway construction can range from 54,000 to 60,000 tons. However, the extraction of natural aggregates from quarries and riverbeds has become increasingly restricted because of heightened environmental protection measures [6]. This has created a critical supply shortage of natural aggregates, posing a significant challenge to the sustainability of highway infrastructure development.

Policymakers in China have acknowledged the significance of aggregate resources and emphasized the need to actively utilize industrial by-products, such as tailings, tunnel slag, and construction waste, as substitutes for natural resources to develop manufactured sand and other aggregate products [7,8,9]. Consequently, the incorporation of tunnel waste slag as an alternative resource for the production of cement-stabilized aggregate base courses in highway engineering can offer a practical and sustainable solution [10]. Tunnel waste slag, produced in substantial quantities during the construction of highways and railways in mountainous regions, holds the potential for recycling and reutilization, effectively addressing both the environmental challenges related to its disposal and the scarcity of natural aggregates [11]. The utilization of tunnel waste slag in highway construction contributes to a circular economy, fostering resource efficiency and mitigating the expenses and environmental ramifications associated with the production and transportation of primary materials [12]. Furthermore, the incorporation of waste materials from tunnel excavations aids in conserving natural resources for posterity [13].

Researchers have been actively exploring the potential applications of tunnel waste slag and investigating its suitability as a construction material [14]. Wang et al. [15] conducted a series of experimental studies to examine the feasibility of using tunnel waste slag as concrete aggregates. Their results showed that with the adoption of appropriate technical measures, tunnel waste slag can be successfully utilized as concrete aggregates, and some high-quality tunnel waste slag can even be applied in pumped concrete applications. Voit et al. [16] argued that the lack of adequate understanding and awareness of tunnel waste slag among practitioners has led to its limited application in construction projects. However, the researchers pointed out that hard tunnel boring machine (TBM) slag, if it meets the standard requirements for building aggregates, can be used in highway pavement concrete and structural concrete [17,18]. Sandanayake et al. [19] reviewed the importance of recovering and recycling the waste materials generated during tunnel excavation. By examining the petrographic features of waste materials and assessing the potential for alkali-aggregate reactions, Song et al. [20] conducted an initial quality assessment of tunnel waste slag. They found that the TBM excavation method is prone to generating larger and flatter aggregate particles, which are better suited for use as concrete aggregates. Li et al. [21] suggested that with the adoption of appropriate technical measures, tunnel waste slag can be utilized as fill material for highway or railway embankments or effectively combined with cement (or asphalt) for various engineering applications. This approach can help avoid the ecological damage caused by the traditional practice of establishing numerous quarries and disposal sites, while also bringing significant economic benefits. In a study on the utilization of tunnel waste slag for highway subgrade construction, Riviera et al. [22] conducted extensive experiments and found that tunnel waste slag, when used as fill material for the subgrade layer, meets all the required specifications and can be successfully applied in practice. Mlinar et al. [23] investigated the issue of tunnel waste slag storage in mountainous highway projects and proposed an innovative solution of using slag as a substrate for slope greening, thereby achieving the circular utilization of the resources. The existing research has primarily focused on analyzing the various approaches to utilizing tunnel waste slag and evaluating the performance of these materials [24,25]. Studies have gradually moved from theoretical investigations to the application of tunnel waste slag in a limited number of engineering projects [26]. However, there is still a lack of standardized guidelines and comprehensive technical guidance for the practical implementation of tunnel waste slag in highway engineering applications.

To provide a systematic framework for the utilization of tunnel waste slag in highway engineering, this study aimed to conduct a comprehensive evaluation of the physical and mechanical properties of tunnel waste slag and explore its potential for utilization in cement-stabilized aggregate base courses. This study involved the characterization of the physical and mechanical properties of the parent rock (tunnel waste slag) and the recycled aggregates derived from it, encompassing parameters such as uniaxial compressive strength, ultrasonic wave velocity, and morphological attributes. Furthermore, this study evaluated the performance of cement-stabilized aggregate base mixtures incorporating recycled aggregates from tunnel waste slag, with a focus on their unconfined compressive strength and splitting strength at various curing durations. A correlation analysis was performed to establish a relationship between the properties of the original rock and the mechanical behavior of the cement-stabilized aggregate base.

The findings of this study provide technical guidance and recommendations for the practical implementation of tunnel waste slag in the construction of cement-stabilized aggregate base courses for highway engineering applications. It is important to note that this study is confined to the utilization of tunnel waste slag generated from a specific highway construction initiative in Jiangxi Province, China. While the insights and conclusions drawn from this research may be transferrable to similar contexts, further investigation is warranted to assess their applicability to other regions or types of tunnel waste slag.

The structure of this paper is as follows: Section 2 presents the materials and methods employed in this study, including the detailed characterization of the tunnel waste slag mother rock and the production of the recycled aggregates, as well as the experimental procedures for evaluating the properties of the cement-stabilized mixtures. Section 3 discusses the results obtained from the various tests and analyses, exploring the relationships among the mother rock properties, the characteristics of the recycled aggregates, and the mechanical performance of the cement-stabilized mixtures. Finally, Section 4 summarizes the key conclusions drawn from this study and highlights the potential implications and future research directions.

## 2. Materials and Methods

### 2.1. Materials

The tunnel waste slag used in this study was obtained from the excavation of the SSA section of the Yisui Expressway project in Jiangxi Province, China. The tunnel waste slag was composed of gneiss rock. Gneiss is a metamorphic rock consisting primarily of feldspar and quartz, with a medium- to coarse-grained crystalline structure and gneissic foliation [27].

**Figure 1:**
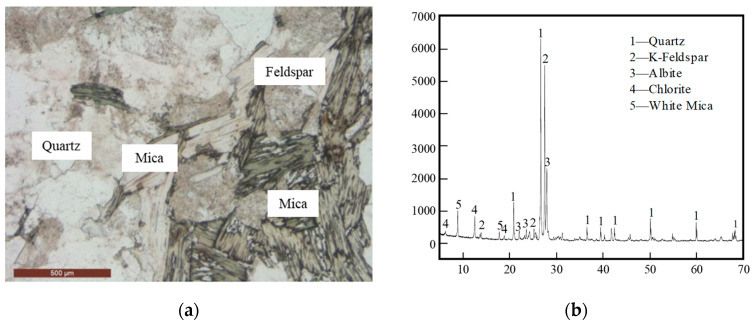
Characteristics of the gneiss rock sample: (**a**) apparent image and (**b**) XRD pattern.

The gneiss samples obtained from the crushing plant were cut into small blocks measuring approximately 3 cm on each side and subsequently prepared into thin sections with a thickness of around 30 μm. These thin sections were then examined and studied, utilizing a Leica DM2700 P polarized light microscope (Leica Microsystems, Wetzlar, Germany), with the outcomes depicted in Figure 1a. The analysis revealed that the gneiss displays a light gray hue with a granular schistose structure and gneissic foliation. The primary mineral constituents comprise quartz, feldspar, a minor quantity of biotite, and garnet. The chemical composition of the gneiss is detailed in Table 1, wherein SiO_2_ and Al_2_O_3_ constitute over 86% of the composition, followed by K_2_O and Na_2_O.

Furthermore, an XRD analysis of the gneiss samples was carried out employing a Rigaku D/MAX 2500 X-ray diffractometer. The procedure involved initial grinding of the samples to a particle size below 75 μm to ensure uniformity. Cu Kα radiation served as the radiation source during the measurement, with the voltage set at 40 kV and the current at 40 mA. The scanning range spanned from 5° to 80°, with a step size of 0.02°. Subsequently, the XRD spectra were scrutinized using Jade v6.5 software, and the findings are illustrated in Figure 1b. The results confirm the mineral composition of the samples to consist of quartz, potassium feldspar, and sodium feldspar, along with minor quantities of chlorite and muscovite.

**Figure 2:**
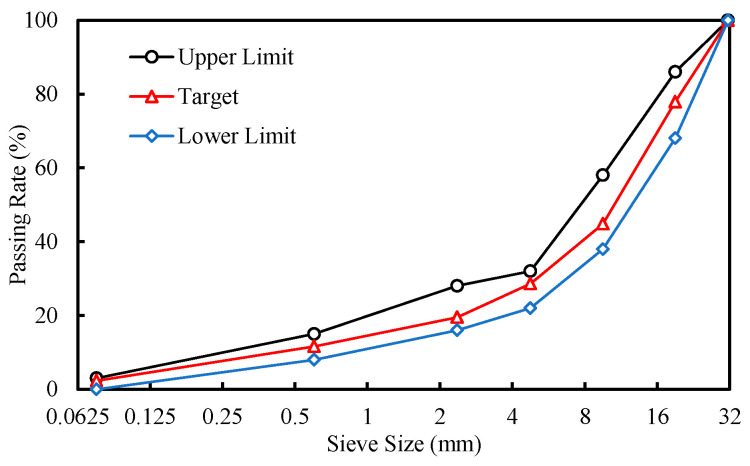
Grading curve of cement-stabilized crushed stone.

The basic physical properties of the recycled aggregates produced from the tunnel waste slag, such as apparent relative density, water absorption, crushing value, clay content, and flakiness index, were determined according to ASTM standards, as shown in Table 2. The recycled aggregates were graded into four fractions as follows: 20–30 mm, 10–20 mm, 5–10 mm, and 0–5 mm. The gradation of the cement-stabilized crushed stone mixture is presented in Figure 2. The cement used in this study was a P.O. 42.5 Portland cement (Jiangxi Wannianqing Cement Co., Ltd., Nanchang, China)with an initial setting time of 206 min and a final setting time of 260 min.

### 2.2. Recycled Aggregate Production Process

Prior to the crushing process, the tunnel waste slag underwent initial screening to eliminate stones and impurities with diameters exceeding 50 mm, such as soil, mud, vegetation, and other debris, in order to guarantee the cleanliness of the material. For oversized slag blocks with diameters surpassing 2 m, manual drilling and splitting techniques were employed to reduce them to smaller dimensions not exceeding 500 mm, thereby facilitating subsequent crushing operations.

**Figure 3:**
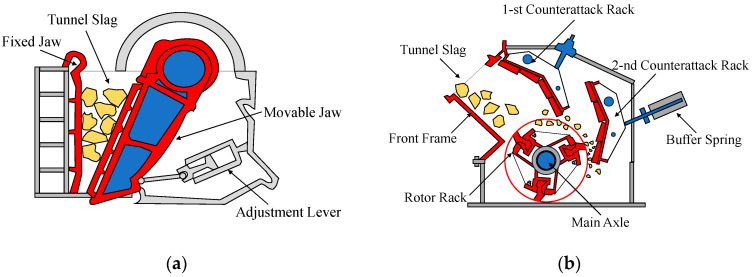
Devices of the tunnel waste slag crushing process: (**a**) jaw crusher; (**b**) impact crusher.

The crushing procedure entailed the utilization of a jaw crusher followed by an impact crusher, as depicted in Figure 3. During the primary crushing phase, the jaw crusher was utilized to fragment the coarse aggregates into smaller dimensions. The movable jaw plate advanced towards the fixed jaw plate at a consistent speed of 250 rpm, maintaining a preset crushing gap of 100 mm to ensure the aggregates were resized to dimensions suitable for the subsequent stage (below 100 mm). Subsequently, the aggregates, by post-primary crushing, were conveyed to the impact crusher for further comminution. The rotor speed of this equipment was configured at 1500 rpm, propelling the aggregates towards the impact plate at high velocity for further fragmentation. The spacing between the impact plate and the rotor was calibrated to 50 mm to regulate the final particle size, guaranteeing that the resulting aggregates ranged from 10 mm to 30 mm in diameter. This size range was selected to align with engineering specifications, ensuring uniformity in aggregate size and suitability for subsequent processing or direct application.

### 2.3. Test Methods

The test methods used in this study included the uniaxial compressive strength of the parent rock, ultrasonic wave velocity, morphological characteristics of the recycled aggregates, unconfined compressive strength, and splitting strength. These methods are described in detail in the following subsections.

#### 2.3.1. Uniaxial Compressive Strength of Parent Rock

**Figure 4:**
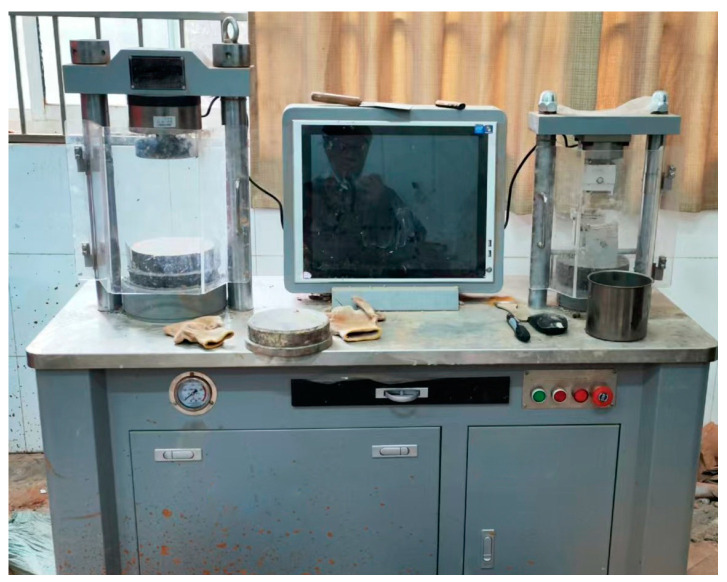
Schematic diagram of the single-axis compression tester.

A uniaxial compression test was carried out by the test instrument shown in Figure 4. The uniaxial compressive strength and softening coefficient of the parent rock were evaluated. Cube-shaped rock specimens with a side length of 50 mm were prepared and tested under both oven-dried and saturated conditions at a loading rate of 0.5 MPa/s until failure. The softening coefficient was calculated as the ratio of the average saturated uniaxial compressive strength to the average oven-dried uniaxial compressive strength.

#### 2.3.2. Ultrasonic Wave Velocity Test

An HS-YT302C rock ultrasonic wave velocity tester, HS-PD100K longitudinal wave transducers, and an HS-ZJ200 ultrasonic testing frame from Xiangtan City Tianhong electronics research Institute, Xiangtan, China, were used to measure the ultrasonic wave velocities of the rock specimens. The rock samples were placed in the testing frame, and a constant pressure of 0.05 MPa was applied to the specimen surfaces before the ultrasonic test. The test parameters were set to a sampling rate of 0.2 μs, a sampling byte of 0.5 K, a pulse width of 2 μs, an attenuation of 20, and a calibration time of 3.39 μs, with a transmitter–receiver voltage of 250 V.

#### 2.3.3. Morphological Characteristics of Recycled Aggregates

Image-Pro Plus v6.0 image processing software was used to analyze the two-dimensional shape parameters of the recycled aggregates, including the maximum diameter, minimum diameter, perimeter, convex hull perimeter, and equivalent ellipse perimeter. Three shape parameters were then calculated to characterize the morphological features of the recycled aggregates, i.e., the aspect ratio, roughness, and angularity, as defined in Equations (1)–(3).

(1) $$Aspect=\frac{D_{\max}}{D_{\min}}$$

(2) $$Roughness=\frac{L}{L_{c}}$$

(3) $$Angularity=\frac{L_{c}}{L_{e}}$$

where *D*_max_ is the maximum diameter of the aggregate particles; *D*_min_ is the minimum diameter of the aggregate particles; *L* represents the perimeter of the aggregate particles; *L_c_* denotes the convex hull perimeter of the aggregate particles; and *L_e_* signifies the equivalent ellipse perimeter of the aggregate particles.

#### 2.3.4. Unconfined Compressive Strength Test

**Figure 5:**
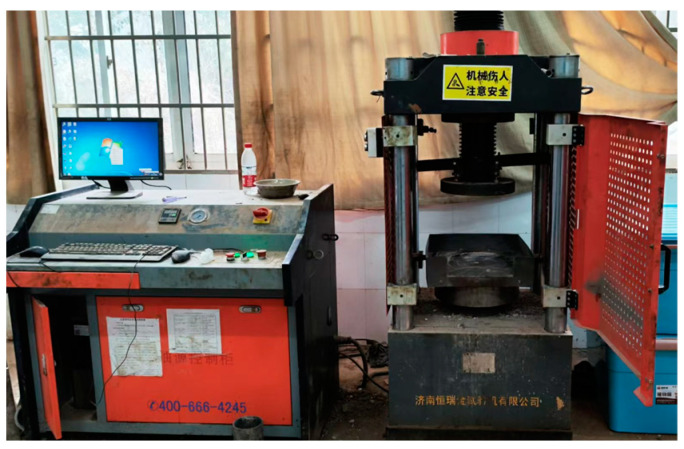
Schematic diagram of the unconfined compression tester.

A uniaxial compression test was carried out by the test instrument shown in Figure 5. Cylindrical specimens with a diameter of 150 mm and a height of 150 mm were prepared for the unconfined compressive strength test. The specimens were sealed in plastic film and cured in a temperature-controlled (20 °C ± 2 °C) and high-humidity (≥95%) chamber. On the last day of curing, the specimens were submerged in water with the water level approximately 2.5 mm above the specimen top. After 24 h of water soaking, the specimens were removed, the surface water was blotted, and the mass and height were measured. The specimens were then placed in a universal testing machine and loaded at a rate of 1 mm/min until failure. The unconfined compressive strength (*R_c_*) was calculated using Equation (4).

(4) $$R_{c}=\frac{P}{A}$$

where *P* represents the maximum pressure at failure of the specimen and *A* denotes the cross-sectional area of the specimen.

#### 2.3.5. Splitting Tensile Strength Test

Based on the ASTM C496 standard [28], the splitting strength (*R_i_*) of the cement-stabilized crushed stone was determined using the split tensile test. Cylindrical specimens with a diameter of 150 mm were tested using an MTS testing machine (MTS Systems Corporation, Eden Prairie, MN, USA). The specimens were placed horizontally between the loading platens, and a compressive load was applied at a rate of 1 mm/min until failure. The splitting strength was calculated using Equation (5).

(5) $$R_{i}=\frac{2P}{\pi dh}\left(\sin2\alpha -\frac{a}{d}\right)$$

where *α* the central angle to the half-strip width; *d* denotes the diameter of the specimen; *a* signifies the width of the pressure strip; and *h* indicates the height of the specimen after water immersion.

#### 2.3.6. Correlation Analysis Method

To enhance the comprehension of the interplay between the physical and mechanical characteristics of cement-stabilized crushed stone materials utilizing tunnel waste slag, this study focused on parameters such as saturated rock strength, shape attributes (including the aspect ratio, roughness, and angularity), 7-day unconfined compressive strength, and 90-day splitting tensile strength for correlation examination. Subsequently, the Pearson correlation coefficient method was applied to evaluate the linear relationships among these parameters. The data processing and correlation analysis were executed using MATLAB v8.0 software. To ensure the robustness of the outcomes, the data underwent standardization to mitigate the impact of dimensional variations on the correlation analysis.

## 3. Results and Discussion

### 3.1. Strength Characteristics of the Parent Rock

**Figure 6:**
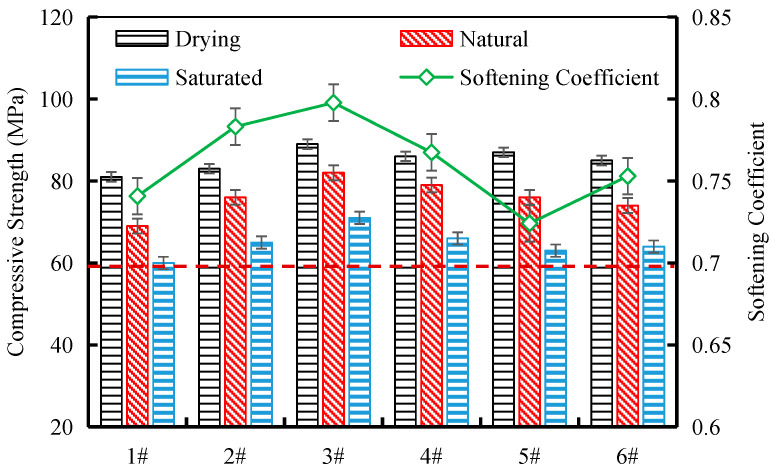
Uniaxial compressive strengths of the tunnel waste slag parent rock samples.

As shown in Figure 6, the uniaxial compressive strengths of the tunnel waste slag parent rock samples from the six sampling locations exhibited the following trend: oven-dried state > natural state > saturated state. The differences in compressive strength among the different sampling locations were relatively small, which can meet the reliability requirements for engineering materials. Taking the oven-dried uniaxial compressive strength as an example, the lowest value was 81 MPa for the sample from location 1#, and the highest value was 89 MPa for the sample from location 3#. The engineering requirement for the compressive strength of the parent rock for manufactured sand is generally not less than 60 MPa. Therefore, all tunnel waste slag samples used in this study met the engineering requirements. The softening coefficients of the tunnel waste slag ranged from 0.72 to 0.80, indicating that the water content in the rock has a significant impact on its uniaxial compressive strength. This is mainly because the filling minerals and rock debris in the rock mineral composition may contain clay minerals, which can undergo dissolution when saturated, leading to a decrease in the saturated compressive strength of the rock.

### 3.2. Ultrasonic Wave Parameters

**Figure 7:**
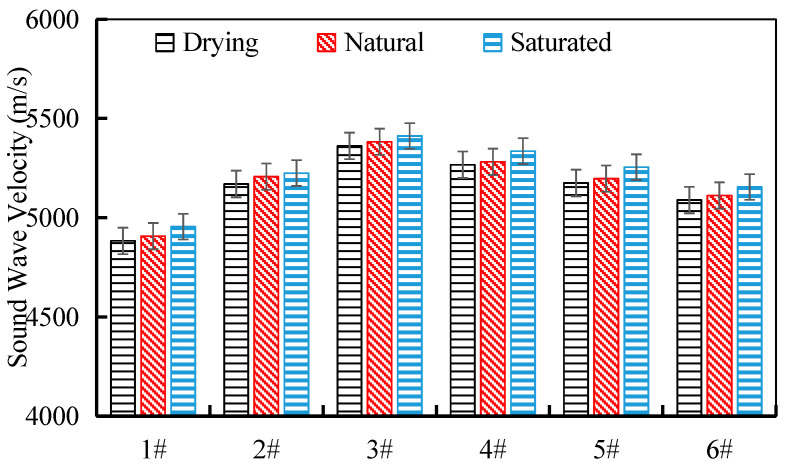
Ultrasonic wave velocities of the tunnel waste slag parent rock samples.

Figure 7 shows the test results of the ultrasonic wave velocities of the tunnel waste slag parent rock in the oven-dried, natural, and saturated states. Overall, the ultrasonic wave velocities of the tested tunnel waste slag parent rock samples exhibited the following trend: oven-dried state < natural state < saturated state. The difference in wave velocity between the saturated and oven-dried states was the largest for sample 5# at 80 m/s. The change in water content of the tunnel waste slag parent rock has a significant impact on the ultrasonic wave velocity, as the wave propagation speed in different media varies. After the sample is saturated, the pores are filled with water, whose density is greater than that of air, resulting in a higher wave propagation speed in water than in air. The absorption water content and internal porosity of the tunnel waste slag parent rock are closely related, and the higher the porosity and the more free water absorbed, the higher the degree of saturation, indicating that the internal porosity and moisture content of the tunnel waste slag parent rock are the key factors affecting the changes in ultrasonic wave velocity.

**Figure 8:**
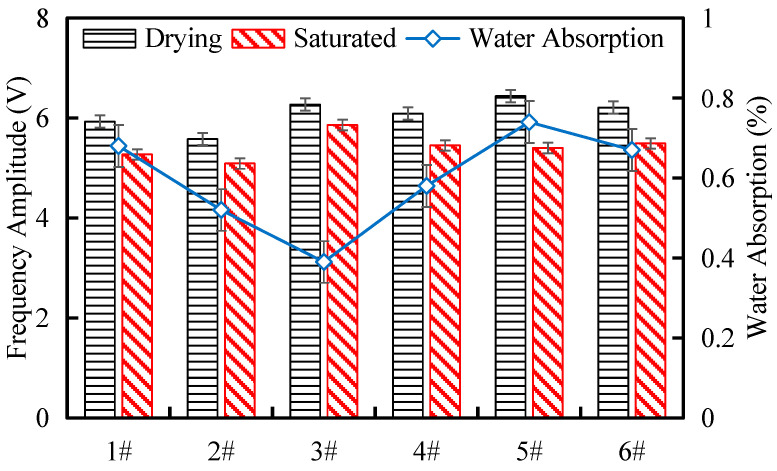
Main frequency amplitudes of the tunnel waste slag parent rock samples.

Figure 8 shows the changes in the main frequency amplitude of the tunnel waste slag parent rock under saturated and oven-dried conditions. It can be seen that the main frequency amplitude of the ultrasonic wave is highly sensitive to changes in the internal water content of the sample. The main frequency amplitude of the tunnel waste slag parent rock in the saturated state is lower than that in the oven-dried state, with the maximum difference in the main frequency amplitude between the saturated and oven-dried states being 1.04 V. This trend is negatively correlated with the softening coefficient. This indicates that the absorption of the main frequency signal energy is enhanced when the tunnel waste slag parent rock is saturated, and the free water in the pores has a significant influence on the main frequency amplitude of the ultrasonic wave. This further reveals the influence of pore water and the internal structure on ultrasonic wave signals, which is helpful for accurately evaluating rock characteristics using wave parameters.

**Figure 9:**
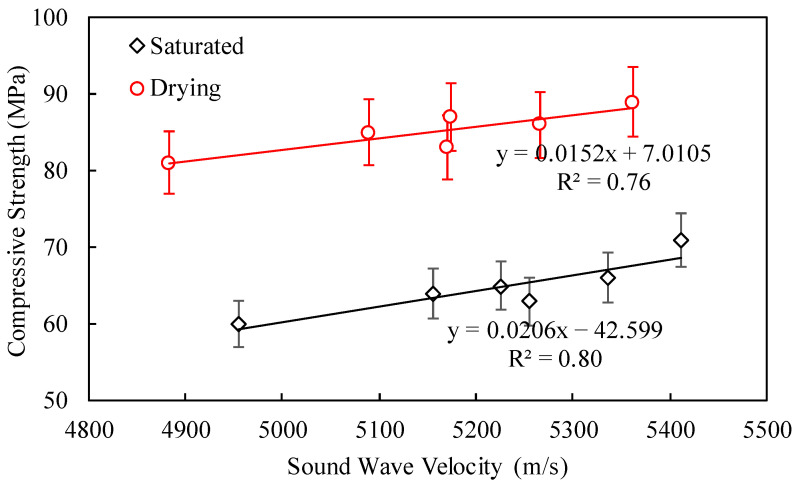
Relationship between ultrasonic wave velocity and saturated compressive strength.

Based on the above discussion, it is known that water has a significant effect on the strength of rocks, and there are differences in ultrasonic wave velocities under different saturation conditions. At the same time, the ultrasonic wave velocity and the saturated uniaxial compressive strength of the rock exhibit similar changing trends, and the influence of water on the strength of the rock is reflected in the ultrasonic wave velocity. Therefore, a prediction model for the compressive strength of rocks based on ultrasonic wave velocity was established. Figure 9 shows the relationship between the ultrasonic wave velocity and the saturated compressive strength of the tunnel waste slag parent rock. The results show that the ultrasonic wave velocities of the tunnel waste slag correspond to the range of 4800–5500 m/s. In the saturated or oven-dried state, the compressive strength of the tunnel waste slag parent rock is linearly related to the ultrasonic wave velocity, with correlation coefficients of 0.80 and 0.76, respectively. Therefore, in actual engineering practice, the compressive strength of the tunnel waste slag parent rock can be predicted by testing its ultrasonic wave velocity, thereby quickly screening out parent rock that meets the engineering requirements for the production of recycled aggregates.

### 3.3. Morphological Analysis of Recycled Aggregates

**Figure 10:**
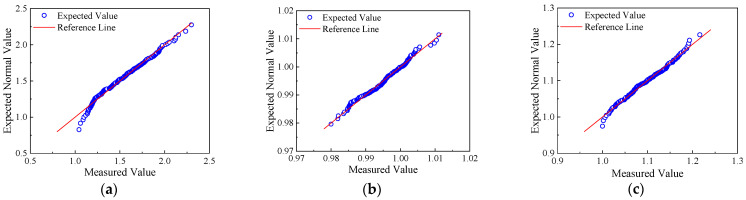
Normal distribution prediction results for three shape parameters: (**a**) aspect, (**b**) roughness, and (**c**) angularity.

The distribution characteristics of the shape parameters of the recycled aggregates from the six tunnel waste slag sampling points should satisfy the normal distribution. Taking the 1# sampling point as an example, Figure 10 shows the scatter plots and normal distribution prediction results of the three shape parameters. Each group of recycled aggregates had a sample size of 200. The results showed that the distribution of the morphological characteristic indicators of the tunnel waste slag recycled aggregates satisfies the normal distribution, which is consistent with the conclusions of previous studies. Similarly, the shape characteristics of the recycled aggregates from the other five sampling points also satisfy the normal distribution. Table 3 lists the mean and standard deviation of the normal distribution of the shape indices of the recycled aggregates from different sampling points.

The results in Table 3 show that the normal distribution means of the aspect of the recycled aggregates are between 1.5509 and 1.6216, the means of roughness are between 0.9915 and 0.9955, and the means of angularity are between 0.0461 and 0.0497. By comparing the shape parameter characteristics with the compressive strength of the parent rock, it can be seen that the recycled aggregates with higher parent rock strength have higher aspect and roughness values and lower angularity values. This indicates that the morphological characteristics of the recycled aggregates are closely related to the strength of the parent rock, and the recycled aggregates with higher parent rock strength tend to have more favorable shape characteristics. Therefore, the shape parameters of recycled aggregates can be used as an important index to evaluate the quality of recycled aggregates.

### 3.4. Compressive Strength of Cement-Stabilized Crushed Stone

Unconfined compressive strength is a crucial parameter for evaluating the performance of cement-stabilized crushed stone in highway engineering applications. In this study, the unconfined compressive strength of the cement-stabilized crushed stone specimens was evaluated at different curing ages of 7 days, 14 days, 28 days, and 90 days.

In addition to the unconfined compressive strength tests, the compaction characteristics of the cement-stabilized crushed stone were also investigated. Standard Proctor compaction tests were conducted in accordance with relevant standards to determine the maximum dry density and optimum moisture content of the material. The results of the compaction tests at different cement contents are presented in Table 4. It can be observed that as the cement content increased from 3.5% to 5.5%, the maximum dry density increased from 2.379 g/cm^3^ to 2.432 g/cm^3^, while the optimum moisture content slightly increased from 4.9% to 5.4%. The improved compaction characteristics with higher cement content are attributed to the enhanced binding and lubricating effects provided by the cement paste.

**Figure 11:**
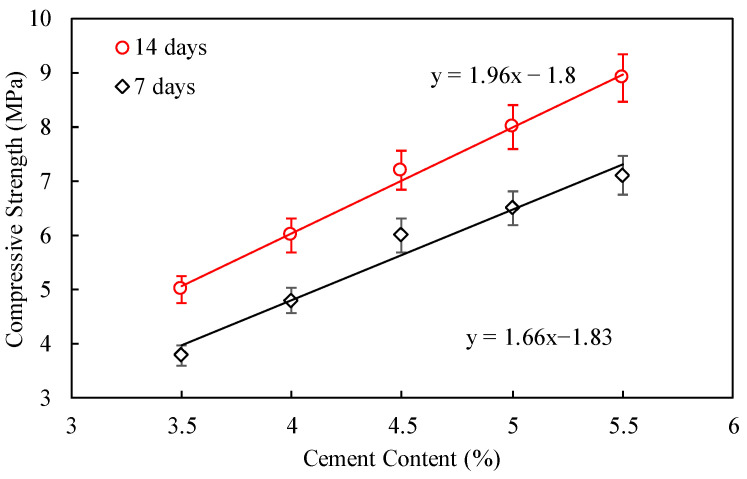
Relationships between cement content and unconfined compressive strength.

Based on the results of the compaction tests, the optimal moisture content was determined for preparing cylindrical specimens of cement-stabilized crushed stone at different cement contents. Figure 11 illustrates the influence of various cement contents on the unconfined compressive strength of specimens at 7 days and 14 days. Overall, similar to the compaction test results, the unconfined compressive strength at 7 days and 14 days exhibits a linear increase with an increase in cement content. Specifically, for each 1% increase in cement content, the unconfined compressive strength at 7 days increases by approximately 1.66 MPa, while the strength at 14 days increases by 1.96 MPa. For highway subbase layers, the required design compressive strength at 7 days is generally around 4.5 MPa, suggesting that the cement content should not be less than 4.0%. Taking into consideration cost reduction, cost savings during actual construction, and the prevention of base cracking due to cement shrinkage, a cement dosage of 4.0% was determined as the target cement content for this mix design.

**Figure 12:**
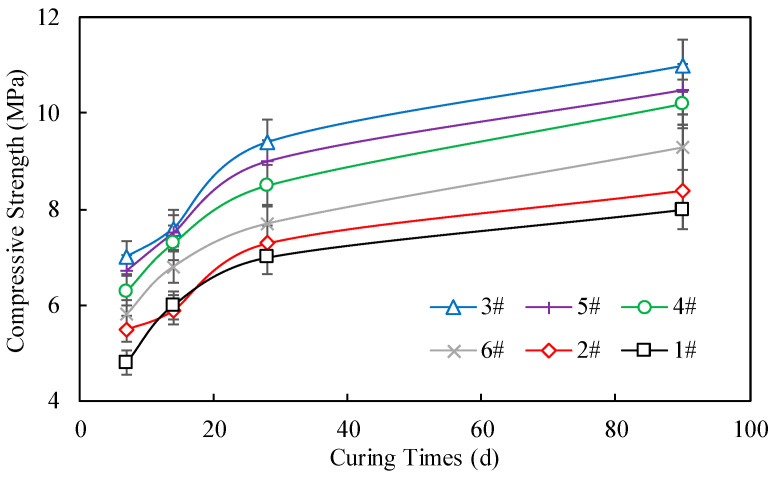
Unconfined compressive strengths of cement-stabilized crushed stone from different sampling locations.

The results of the unconfined compressive strength tests of samples from different locations are presented in Figure 12. It can be observed that the unconfined compressive strength of the cement-stabilized crushed stone made from the tunnel waste slag recycled aggregates increased significantly as the curing time increased. For the sample from location 3#, the unconfined compressive strength increased from 7.0 MPa at 7 days to 9.4 MPa at 28 days and further increased to 11.0 MPa at 90 days. This indicates that the cement-stabilized crushed stone made from the tunnel waste slag recycled aggregates can develop sufficient early-age and long-term strength to meet the requirements for highway base/subbase applications.

For tunnel waste slag samples from locations 1# to 6#, the proportions of the strength formed in the first 7 days of the cement-stabilized crushed stone materials to the overall strength at 90 days are as follows: 60%, 65.5%, 63.6%, 61.8%, 63.8%, and 62.4%, respectively. It is evident that more than half of the strength is achieved within the first 7 days. For different parent rocks, the unconfined compressive strength of cement-stabilized crushed stone increases with the curing age, with the sample from location 3# consistently maintaining the highest strength. This could be attributed to the inherently high strength of the parent rock from location 3#. Therefore, when selecting tunnel waste slag as aggregate, priority should be given to parent rocks with higher strength.

The increase in unconfined compressive strength with curing time can be attributed to the continuous hydration and hardening of the cement paste, which binds the recycled aggregates together and enhances the overall compressive strength of the cement-stabilized crushed stone. The recycled aggregates from the tunnel waste slag have a relatively high parent rock strength, as demonstrated by the uniaxial compressive strength tests in Section 3.1. The strong parent rock provides a stable skeleton for the cement-stabilized crushed stone mixture, which is beneficial for the development of compressive strength.

It is also worth noting that the variability in the unconfined compressive strength test results of the cement-stabilized crushed stone is relatively small. The mean coefficient of variation (COV) of the unconfined compressive strength at 7 days, 14 days, 28 days, and 90 days was 5.7%, 4.9%, 4.2%, and 4.0%, respectively. In addition, the Chinese specification JTG/T F20-2015 clearly states that the 7-day compressive strength should be used as the primary index for construction quality control. Referring to both this specification and Figure 10, it is evident that, with the exception of the cement-stabilized crushed stone from site #1—which only meets the heavy traffic standard (4~6 MPa) for motorways—the cement-stabilized crushed stone from the other sites meets the extra-heavy traffic criterion (5~7 MPa) for expressways. This indicates that the recycled aggregates from the tunnel waste slag have consistent quality, which is crucial for ensuring the stability and reliability of cement-stabilized crushed stone in highway engineering applications.

The high and stable unconfined compressive strength of the cement-stabilized crushed stone made from the tunnel waste slag recycled aggregates demonstrates their suitability for use in highway base/subbase layers. The efficient utilization of tunnel waste slag as recycled aggregates not only reduces the consumption of natural aggregates but also contributes to the sustainable development of highway infrastructure construction.

### 3.5. Splitting Strength of Cement-Stabilized Crushed Stone

Splitting tensile strength is an important parameter that reflects the resistance of cement-stabilized crushed stone materials to splitting stresses, which is crucial for their performance in pavement structures. In this study, the splitting tensile strength of the cement-stabilized crushed stone specimens was evaluated at curing ages of 28 days, 60 days, and 90 days.

**Figure 13:**
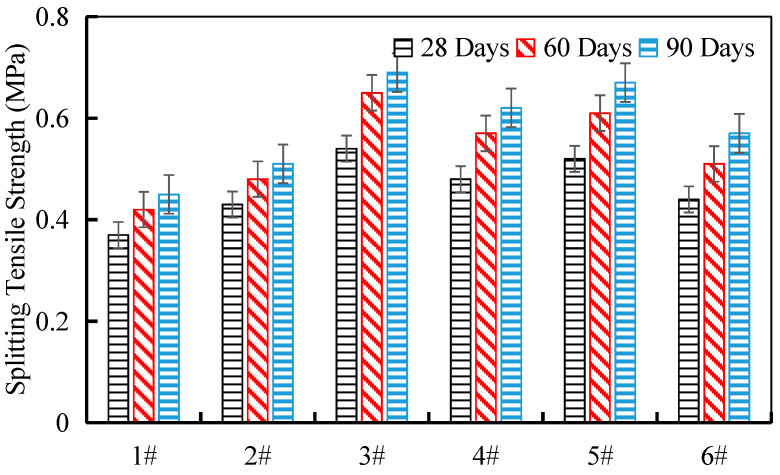
Splitting tensile strength of cement-stabilized crushed stone from different sampling locations.

The results of the splitting tensile strength tests are presented in Figure 13. It can be observed that the splitting tensile strength of the cement-stabilized crushed stone increased with the curing age. From 28 days to 60 days, the splitting tensile strength increased by 13.5%, 11.6%, 20.4%, 18.8%, 17.3%, and 15.9% for the six different tunnel waste slag sources, respectively. The rate of strength gain was more pronounced in the early curing period (28 to 60 days) compared with the later stage (60 to 90 days).

The improvement in splitting tensile strength with curing age can be attributed to the continued hydration of the cement and the formation of hydration products, particularly ettringite, which can effectively fill the voids within the cement-stabilized mixture and provide enhanced tensile resistance. The differences in splitting tensile strength among the tunnel waste slag sources can be correlated to the inherent strength characteristics of the parent rock materials, with the lower-strength samples (e.g., sample 1#) exhibiting lower splitting tensile strength because of their reduced particle resistance to external forces.

The ability to develop adequate splitting tensile strength is crucial for cement-stabilized crushed stone to withstand the tensile stresses induced by traffic loads and environmental effects in pavement structures. The results demonstrate that the cement-stabilized tunnel waste slag materials can achieve satisfactory splitting tensile strength characteristics, making them suitable for use in high-performance pavement applications.

### 3.6. Correlation Analysis of the Strength Characteristics of Cement-Stabilized Crushed Stone

To better understand the relationships between the various physical and mechanical properties of the cement-stabilized crushed stone materials, a correlation analysis was performed. The parameters considered in this analysis include the saturated rock strength, aspect ratio, roughness, angularity, 7-day compressive strength, and 90-day splitting tensile strength. The data used for the correlation analysis is presented in Table 5. This table provides the test results for the different tunnel waste slag sources (samples 1# to 6#) in terms of the measured parameters.

**Figure 14:**
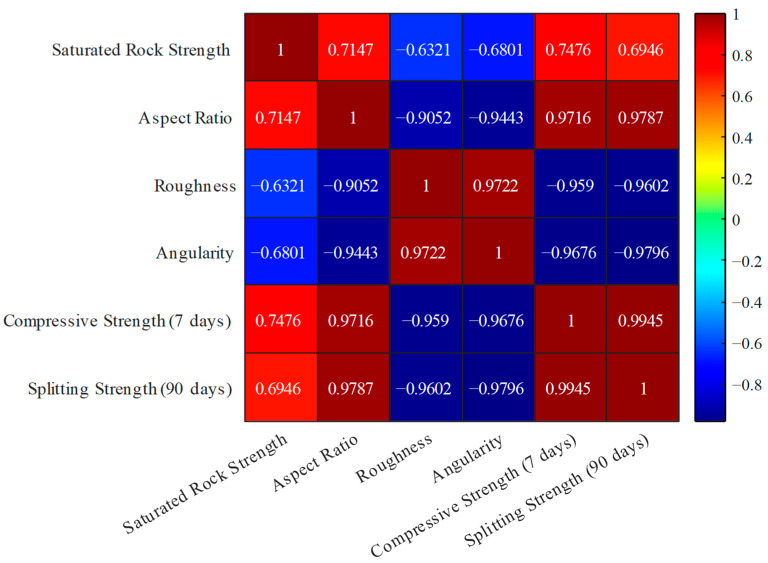
Correlation heat map of the strength characteristics of cement-stabilized crushed stone.

The resulting correlation heat map is shown in Figure 14. The heat map visually represents the correlation coefficients among the different parameters, where the intensity of the color indicates the strength of the correlation. From the heat map, the following key observations can be made:

(1) The saturated rock strength shows a strong positive correlation with the 7-day compressive strength (r = 0.91) and the 90-day splitting tensile strength (r = 0.95). This indicates that the inherent strength of the parent rock material is a dominant factor in determining the overall mechanical properties of the cement-stabilized crushed stone.
(2) The aspect ratio, roughness, and angularity exhibit moderate to strong positive correlations with the compressive and splitting tensile strengths. This suggests that the particle characteristics of the crushed stone can also influence the performance of the cement-stabilized material.
(3) The compressive strength at 7 days shows a high correlation (r = 0.95) with the splitting tensile strength at 90 days. This implies that the early-age compressive strength can be a good predictor of the long-term tensile performance of cement-stabilized crushed stone.

The correlation analysis provides valuable insights into the relationships between the various physical and mechanical properties of cement-stabilized crushed stone materials. These insights can be used to optimize the mix design and selection of suitable tunnel waste slag sources to achieve the desired performance characteristics for pavement applications.

## 4. Conclusions

This comprehensive study on the utilization of tunnel waste slag in cement-stabilized aggregate base courses for highway engineering applications led to the following key conclusions:

(1) The tunnel waste slag parent rock exhibited high uniaxial compressive strengths, ranging from 81 MPa to 89 MPa in the oven-dried state, which met the engineering requirements for use as a construction material. The softening coefficients of the parent rock ranged from 0.72 to 0.80, indicating that the water content has a significant impact on its compressive strength.
(2) The ultrasonic wave velocities of the tunnel waste slag parent rock were found to be closely related to their saturated compressive strengths, with correlation coefficients of 0.80 and 0.76 for the saturated and oven-dried states, respectively. This relationship allows for the prediction of rock compressive strength based on its ultrasonic wave velocity, enabling efficient screening of parent rock suitable for the production of recycled aggregates.
(3) The shape parameters of the recycled aggregates, encompassing the aspect ratio, roughness, and angularity, were intricately connected to the strength of the original rock. Aggregates originating from more robust parent rock showcased superior shape attributes, reflecting higher-quality aggregates.
(4) The cement-stabilized aggregate base mixtures incorporating recycled aggregates from tunnel waste slag demonstrated satisfactory mechanical properties, with unconfined compressive strengths ranging from 4.5 MPa to 6.9 MPa and splitting strengths ranging from 0.48 MPa to 0.73 MPa at 28 days of curing. These results confirm the feasibility of utilizing tunnel waste slag as a sustainable alternative to natural aggregates in the construction of highway engineering applications.

The effective integration of tunnel waste slag in cement-stabilized base courses presents a sustainable substitute for natural aggregates, tackling environmental issues and material scarcities in highway construction. These discoveries offer crucial insights for the pragmatic implementation of tunnel waste slag in civil engineering endeavors. Subsequent research should concentrate on the extended performance and resilience of these materials under diverse environmental circumstances, along with exploring the feasibility of broader utilization across various forms of tunnel waste slag.

## Figures and Tables

**Table 1:** Chemical components of the gneiss rock sample.

SiO_2_	Al_2_O_3_	Fe_2_O_3_	CaO	MgO	TiO_2_	Na_2_O	K_2_O	P_2_O_5_	SO_3_	Others
71.71	14.78	1.65	1.43	0.48	0.21	3.44	4.73	0.07	0.09	1.41

**Table 2:** Physical properties of recycled aggregates.

Performance	Unit	Value	Requirement
Apparent relative density	g/cm^3^	2.738	≥2.65
Water absorption	%	2.13	≤3.0
Crushing value	%	21.3	≤26
Clay content	%	0.8	≤2.0
Needle-like content	%	8.29	≤18

**Table 3:** Mean and standard deviation of the normal distribution of recycled aggregate shapes.

Sampling No.	Aspect		Roughness		Angularity	
	Mean	STD	Mean	STD	Mean	STD
1#	1.5509	0.2658	0.9955	0.0058	1.1005	0.0461
2#	1.5754	0.3013	0.995	0.0077	1.0987	0.0547
3#	1.6216	0.463	0.9915	0.0062	1.0507	0.0797
4#	1.612	0.3402	0.9926	0.0083	1.0666	0.0554
5#	1.6146	0.2963	0.9917	0.0055	1.0599	0.0675
6#	1.5971	0.3447	0.9947	0.0049	1.0821	0.0846

**Table 4:** Compaction test results of cement-stabilized crushed stone.

Cement content (%)	3.5	4.0	4.5	5.0	5.5
Optimum moisture content (%)	4.9	5	5.2	5.2	5.4
Maximum dry density (g/cm^3^)	2.379	2.393	2.411	2.427	2.432

**Table 5:** Physical and mechanical properties of cement-stabilized crushed stone.

Sampling No.	Saturated Rock Strength (MPa)	Aspect	Roughness	Angularity	7-Day Compressive Strength (MPa)	90-Day Splitting Strength(MPa)
1#	60	1.5509	0.9955	1.1005	4.8	0.45
2#	65	1.5754	0.995	1.0987	5.5	0.51
3#	71	1.6216	0.9915	1.0507	7	0.69
4#	66	1.612	0.9926	1.0666	6.3	0.62
5#	63	1.6146	0.9917	1.0599	6.7	0.67
6#	64	1.5971	0.9947	1.0821	5.8	0.57

## Data Availability

The raw data supporting the conclusions of this article will be made available by the authors upon request.
